# Draft Genome Sequence of *Myroides* sp. N17-2, a Multidrug-Resistant Bacterium Isolated from Radiation-Polluted Soils

**DOI:** 10.1128/genomeA.01301-17

**Published:** 2017-11-16

**Authors:** Tingting Liu, Liying Zhu, Zhidong Zhang, Ling Jiang, He Huang

**Affiliations:** a College of Food Science and Light Industry, Nanjing Tech University, Nanjing, People’s Republic of China; b College of Biotechnology and Pharmaceutical Engineering, Nanjing Tech University, Nanjing, People’s Republic of China; c College of Chemical and Molecular Engineering, Nanjing Tech University, Nanjing, People’s Republic of China; d Institute of Microbiology, Xinjiang Academy of Agricultural Sciences, Urumqi, Xinjiang, Uyghur Autonomous Region, People’s Republic of China

## Abstract

We report here the 4.29-Mb draft genome sequence of *Myroides* sp. N17-2, a new bacterium isolated from radiation-polluted soils in Xinjiang, Uyghur Autonomous Region, China. The acquisition of its genome will provide valuable information to reveal the relationship between radiation and multidrug resistance.

## GENOME ANNOUNCEMENT

The genus *Myroides*, proposed as belonging to the family *Flavobacteriaceae*, was established with the reclassification of the species *Flavobacterium odoratum* (1). Members of this genus are nonfermenting, Gram-negative, aerobic, and nonmotile bacteria (2) that previously have been discovered only from clinical sources, aquatic environment, grey mullet’s gut, and flesh flies (3–5). A new *Myroides* strain, N17-2, was first isolated from radiation-polluted soils in Xinjiang, Uyghur Autonomous Region, China. The whole-genome sequence of N17-2 may contribute to excavating correlative multidrug resistance genes.

Genomic DNA of strain N17-2 was extracted using the EZNATM yeast DNA spin protocol kit according to the manufacturer’s instructions (Omega Bio-Tek, USA). The original image data were obtained by performing next-generation sequencing on the Illumina HiSeq TM2000 platform (6) and constructing an Illumina paired-end (300-bp) library, which was converted into sequencing data via base calling. The trimmed data were sequenced, generating 214,324 reads (Q20, 98.35%). Ultimately, the clean data were joined by multiple *k*-mer parameters using SOAPdenovo version 2.04 (7) and further optimized by GapCloser verison 1.12 (8).

The draft genome of N17-2 was assembled into 4,501,726 bp with an average G+C content of 34.3% at 345.15-fold coverage (9), harboring 128 scaffolds (>1,000 bp in length) and 143 contigs (>1,000 bp in length). The sizes of the *N*_50_ and *N*_90_ scaffolds were 88,131 bp and 27,624 bp, respectively. Using Barrnap version 0.4.2 and tRNAscan-SE version 1.3.1 software, 89 tRNA and 3 rRNA (5S) regions were identified. The predicted 149 tandem repeat regions (0.85% of the genome) indicate the rearrangements in the genome and the horizontal transfer of genes in bacteria (10). The number of candidate protein-coding sequences, the overall length of which accounted for approximately 82.27% of the genome, was 3,684. In addition, a KEGG metabolic pathway was constructed, and BLASTn searches against the clusters of orthologous groups, eukaryotic orthologous groups (KOG), nonredundant, Swiss-Port, and gene ontology databases were used to annotate the open reading frames of strain N17-2.

Based on the analysis of the genome of *Myroides* sp. N17-2, ~0.18% of the chromosome was predicted to encode genes involved in multidrug resistance, namely, the multidrug resistance proteins EmrY, NorM, and YkkD, the multidrug transporters AcrB and MatE, the multidrug export protein EmrA, and even the multidrug resistance ATP-binding cassette (ABC) transporter and multidrug resistance-like ATP-binding protein MdlA, which provides a favorable profile for verifying the multidrug resistance characteristics of N17-2 from a radiation-polluted environment, as well as a foundation for a deeper understanding of the relationship between radiation and multidrug resistance. Moreover, the genome sequence of N17-2 will increase the breadth of information pertaining to members of the genus *Myroides*.

### Accession number(s).

This whole-genome shotgun project has been deposited at DDBJ/ENA/GenBank under the accession number NSGJ00000000. The version described in this paper is the first version, NSGJ01000000.
